# CF-YOLOX: An Autonomous Driving Detection Model for Multi-Scale Object Detection

**DOI:** 10.3390/s23083794

**Published:** 2023-04-07

**Authors:** Shuiye Wu, Yunbing Yan, Weiqiang Wang

**Affiliations:** School of Automobile and Traffic Engineering, Wuhan University of Science and Technology, Wuhan 430065, China

**Keywords:** object detection, YOLOX, attention module, object-contextual feature fusion

## Abstract

In self-driving cars, object detection algorithms are becoming increasingly important, and the accurate and fast recognition of objects is critical to realize autonomous driving. The existing detection algorithms are not ideal for the detection of small objects. This paper proposes a YOLOX-based network model for multi-scale object detection tasks in complex scenes. This method adds a CBAM-G module to the backbone of the original network, which performs grouping operations on CBAM. It changes the height and width of the convolution kernel of the spatial attention module to 7 × 1 to improve the ability of the model to extract prominent features. We proposed an object-contextual feature fusion module, which can provide more semantic information and improve the perception of multi-scale objects. Finally, we considered the problem of fewer samples and less loss of small objects and introduced a scaling factor that could increase the loss of small objects to improve the detection ability of small objects. We validated the effectiveness of the proposed method on the KITTI dataset, and the mAP value was 2.46% higher than the original model. Experimental comparisons showed that our model achieved superior detection performance compared to other models.

## 1. Introduction

With the development of convolutional neural networks [1], artificial intelligence technology has achieved great results and is applied in various fields, such as medicine [2], multimedia [3], and the field of autonomous driving.

Object detection is a critical part of achieving autonomous driving. The basic strategy of the traditional visual object detection algorithm is to first select the interested image region from the image, then extract the corresponding image features from the region, and then finally the extracted features are fed into the classifier. Such algorithms involve two aspects: feature extraction methods, such as the sliding window method [4], SIFT [5], HOG [6], etc., where the features are designed manually; and classifiers, such as SVM classifiers [7], Adaboost classifiers [8], etc. Because the traditional method needs to pre-select the region, it will make the model time-consuming. These models have limitations due to the limited functional capabilities of manual design and the diversity and complexity of the environment.

The progress made in deep learning has been instrumental in driving the growth of computer vision. It is crucial in the domains of object classification [9,10,11], detection [12,13], segmentation [14], tracking [15], etc. The AlexNet model won the ImageNet dataset image classification competition, giving the object detection field a new development direction and benefiting research in autonomous driving. Deep learning network models are trained using many labeled data to find the optimal values while saving the training parameters. Object detection based on deep learning can be categorized into single-stage and two-stage methods. When single-stage algorithms are not developed maturely, two-stage algorithms such as R-CNN [16], Fast R-CNN [17], and Faster R-CNN [18] have higher detection performance. However, FPS is insufficient to qualify as realtime. The model represented by SSD [19] and YOLO [20] first inputs the images directly into the model, then carries out a series of convolution operations, and then conducts regression detection of the box position and object category. These models have fast detection speed but insufficient recognition accuracy. Today, single-stage algorithms have higher detection speed and accuracy and are more suitable to be equipped in complex scenes.

However, the existing models are not optimal in specific scenarios and, thus, need to be adjusted accordingly. Wang et al. [21] proposed a network that can mitigate the impact of rainfall. They accomplished this by devising loss function components that cater to the distinct characteristics of the various subnetworks. Tan et al. [22] proposed a composite scaling method to adapt to different environments, which was trained on the COCO dataset to obtain the optimal mAP at that time. Wong et al. [23] proposed a compact deep convolutional neural network that can be deployed to embedded devices to accommodate the detection speed requirements in different scenarios. Nayereh et al. [24] proposed an efficient hybrid approach of fuzzy and NMS algorithms that can raise the average detection accuracy of the model for vehicle objects. Stefano et al. [25] proposed a new sampling-free uncertainty estimation method, which can effectively cope with unprecedented vehicle driving scenarios and generalize better than the previous methods.

Choi et al. [26] proposed Gaussian YOLOv3 to reduce the localization error of the vehicle objects in autonomous driving. Li et al. [27] developed a stepwise domain adaptive YOLO (S-DAYOLO) framework to improve the object detection performance in various domain shift scenarios for autonomous driving. Li et al. [28] proposed an improved lightweight YOLOv5s network with a higher detection speed and enhanced object localization capabilities. Other new object detection algorithms consider both speed and accuracy [29,30]. But these models are not necessarily suitable for detecting multi-scale objects in complex scenes.

In this paper, we propose a network model based on YOLOX [31] named CF-YOLOX, which has good detection performance for multi-scale objects in autonomous driving scenarios. The main innovations and contributions of this paper are as follows: (1) To further enhance the feature information extraction ability of the model and highlight salient feature information, we propose a CBAM-G module, which can increase the attention weight of salient features. (2) To improve the ability to detect multi-scale objects, we propose an object-contextual feature fusion module to fuse multi-scale object feature information and act on the prediction network to improve the detection effect. (3) To enhance the detection ability of small objects, we propose an improved IOU-LOSS calculation method, which can enhance the loss of small objects. 

The rest of the paper is as follows: In Section 2, we introduce the theory and framework of the YOLOX algorithm. In Section 3, we elaborate on the method proposed in this paper. In Section 4, the proposed experimental method was used on the KITTI [32] dataset and the BDD100K [33] dataset, and our method is compared with other algorithms. Section 5 is the conclusion.

## 2. Structure and Features of YOLOX

YOLOX combines mature network models and effective training techniques without preparing a priori box and modifies the structure of CSPNet [34] to balance speed and accuracy. The backbone network CSPDarknet differs from YOLOv3 [35] in that it incorporates the Focus structure, compressing the image’s shape, increasing channels, reducing parameters, and improving the inference speed. The SiLU activation function used in the model is non-linear, which can solve the gradient divergence problem when the input is negative and make the convergence speed faster. SPP [36] becomes part of the backbone and pools the images with pooling kernels of different sizes, which can expand the receptive fields. The neck uses the PANet [37] network, which fuses feature maps at different scales and can contain information such as the location, texture, and edges at low levels, along with reliable semantic information at high levels.

In the output stage of traditional networks, the object classification and regression tasks are performed directly on the same feature map, which can lead to conflicting tasks because the classification task considers the feature differences between samples, while the regression task focuses more on the profile features of the object. The decoupled head of YOLOX uses a convolutional kernel of size 1 × 1 to adjust the number of channels of the feature map and then connects two parallel branches. Each branch uses convolutional kernels of size 3 × 3 for the classification and regression tasks, which are combined at the prediction time.

YOLOX follows the Mosaic data enhancement technique and introduces the Mixup enhancement method. The Mixup method mixes two images with RGB values in a particular ratio to construct new training samples and labels by linear interpolation. Constructing new samples can enhance the model’s generalization ability, thus, improving the accuracy rate.

The YOLO series models use Anchor-based detection networks, which are trained to solve for the optimal prior box size to optimize the model, but can only be applied to specific datasets and have poor general performance. In YOLOX, an anchor-free detector is used to predict the coordinates of objects. The regression network predicts the coordinates of the upper left corner of the object bounding box (x, y) as well as its width and height. The classification network predicts the object class, while the prediction network distinguishes between the object and the background regions and uses the object’s centroid as a positive training sample. Each point on the output feature map predicts only one pre-selected box. The prediction result matches the original sample to determine whether it is positive, requiring a suitable label assignment strategy. YOLOX adopts SimOTA as the label-matching method. Firstly, the coordinates and category information of the preselection box and the target box must be obtained. Next, ten candidate boxes corresponding to each object frame are obtained by calculating the IOU value. The cost function can be computed by incorporating both regression and classification losses.

(1) $$c_{ij}=L_{ij}^{cls}+\lambda L_{ij}^{reg}$$

In the formula, $L_{ij}^{cls}$ represents the classification loss between the 𝑖th real object frame and the 𝑗th preliminary screening positive sample prediction frame, $L_{ij}^{reg}$ represents the position regression between the 𝑖th real object frame and the 𝑗th primary screening positive sample prediction frame loss, and *λ* represents the weight coefficient of the position regression loss. Candidate boxes are selected for each object using cost values, and duplicate detection boxes are filtered out using NMS. SimOTA can automatically analyze how many positive samples each actual box should have and automatically decide which feature map of each actual frame should be detected, which is beneficial for datasets with uneven sample distribution.

**Figure 1:**
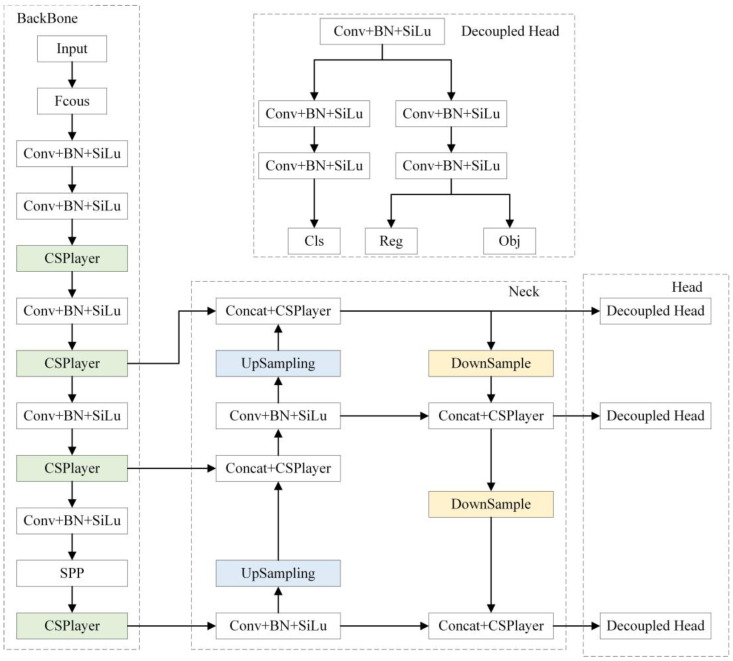
The network structure of YOLOX.

The network’s general architecture is depicted in Figure 1. 

## 3. The Improved YOLOX Network Model

Firstly, this section gives a detailed introduction to the CBAM-G attention module. Next, we detail how the object-contextual feature fusion module works. Then, the improved IOU-LOSS calculation method is explained. Finally, the whole model structure is summarized.

### 3.1. CBAM-G

**Figure 2:**
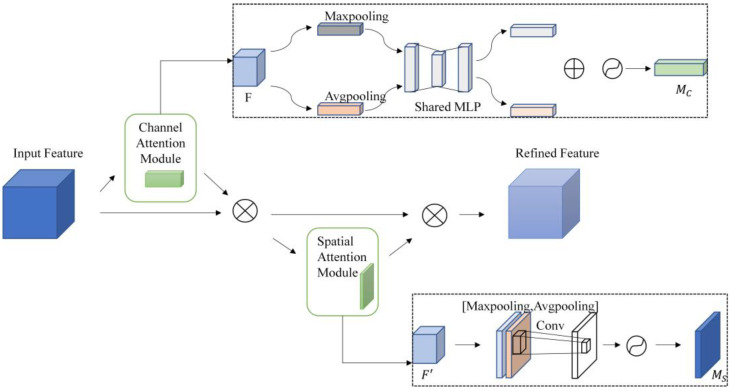
The structure of CBAM.

CBAM [38] has been favored by many researchers for its plug-and-play and significant enhancement since it was proposed in 2018. This module derives the relevance weight matrix for the input feature map in both the channel and spatial dimensions. The matrix is subsequently multiplied with the feature map to acquire the adaptive feature-adjusted feature map. It has almost no effect on the inference operations. Figure 2 shows the architecture of CBAM.

CBAM contains attention modules in both the channel and spatial dimensions. For a given feature map $F\in R^{C\times H\times W}$ that is input to the CBAM module, the channel attention module focuses on the image that is “what” and compresses the spatial dimension of the input feature map. In the channel dimension, the feature map is subjected to global max pooling and mean pooling, resulting in two pooled 1D vectors. Then the 1D vectors are summed after a fully connected layer to obtain the 1D channel attention $M_{C}\in R^{C\times 1\times 1}$, multiplied by the input feature map F to construct a new feature map $F^{\prime }$, represented as follows:

(2) $$M_{C}(F)=\sigma (MLP(AvgPool(F))+MLP(MaxPool(F)))=\sigma (W_{1}(W_{O}(F_{avg}^{C}))+W_{1}(W_{O}(F_{\max}^{C})))$$

In the formula, σ is the sigmoid function, $W_{O}\in R^{C/r\times C}$, $W_{1}\in R^{C\times C/r}$.

$F^{\prime }$ passes through the spatial attention module, focusing on the “where” of the image object. In the spatial dimension, it will go through global max pooling and mean pooling, stack the pooled 2D vectors and then perform a convolution operation to get 2D spatial attention, represented as follows:

(3) $$M_{S}(F)=\sigma (f^{7\times 7}([AvgPool(F);MaxPool(F)]))=\sigma (f^{7\times 7}([F_{avg}^{S};F_{\max}^{S}]))$$

In the formula, $f^{7\times 7}$ is the convolution kernel of 7 × 7; Then the spatial attention is multiplied by the feature map $F^{\prime }$ by element to obtain the final attention output. The whole attention process can be described as follows:

(4) $$F^{\prime }=M_{C}(F)⊗F$$

(5) $$F^{\prime \prime }=M_{S}(F^{\prime })⊗F^{\prime }$$

**Figure 3:**
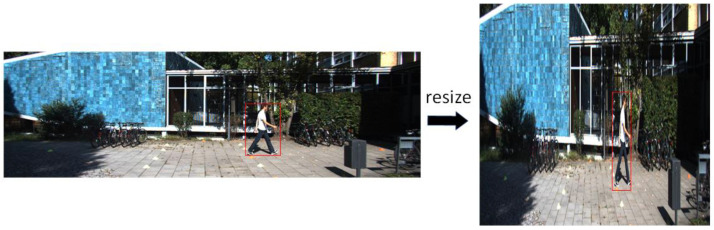
Change of picture object shape.

The feature map is directed to the spatial attention module of CBAM after passing through its channel attention module. This module uses a convolution kernel of 7 × 7 to concentrate on the spatial features. The commonly used camera resolution is mostly larger in width and smaller in height, and the image size is also large in width and small in height. The image shape is similar to a horizontal rectangle. The image aspect resolution of the KITTI dataset for autonomous driving is 375 × 1242, and the picture is a horizontal rectangle. The picture input to the YOLO-S model will be fixed to 640 × 640, and the picture’s aspect ratio will change. The object shape becomes a narrow and high vertical rectangle, deviating from the original object shape size. The shape change of the object after reshaping is shown in Figure 3.

**Figure 4:**
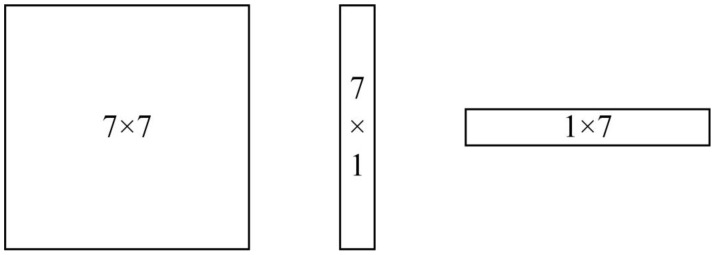
The shapes of convolution kernels of different sizes.

Therefore, the square convolution kernel size of 7 × 7 is not optimal for the feature map, and the extensive convolution range introduces non-object feature information. In this regard, to make the feature extraction fit the original image object information more closely, a convolution kernel with a size of 7 × 1 is used in the CBAM module. The convolution kernel becomes a vertical rectangular shape instead of a square shape. The shapes of convolution kernels of different sizes are shown in Figure 4.

Using a convolution kernel with a size of 7 × 1 to fit the reshaped shape of the original image object does not pay attention to all the information in a large range, which can improve the detection accuracy and model generalization ability to a certain extent. It should be noted that the convolution kernel of size 7 × 1 does not apply to all images. Only images similar in size to 375 × 1242 will work well.

The attention module will effectively enhance the model’s feature extraction capability after focusing on locally important information and suppressing the unimportant information in the image. At the same time, the grouping operation is used to raise the spatial perception of the model and minimize the parameters and computations.

**Figure 5:**
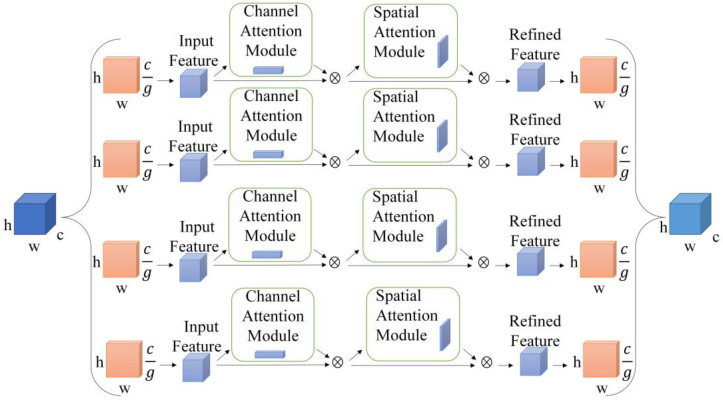
The structure of CBAM-G.

Figure 5 shows CBAM-G. The feature maps are formed into groups after the grouping operation. The size of each group of feature maps is H×W×C/g. The feature maps are passed through the CBAM module for channel and spatial perceptual attention, respectively, to obtain newly grouped feature maps; a stacking process is carried out on every group to generate a feature map that is identical to the initial sized one. 

Without considering the bias term, etc., the calculation quantity formula can be roughly simplified as follows:

(6) $$GFLOS=C_{1}\times C_{2}\times (\frac{H-h}{S_{h}}\times \frac{W-w}{S_{w}})\times (h\times w)$$

The equation involves several variables, $C_{1}\times C_{2}$ denotes the channels of the feature map, and *W* and *H* represent the width and height of the feature map. Additionally, *w* and *h* refer to the width and height of the convolution kernel, while $S_{w}$ and $S_{h}$ denote the step size corresponding to the width and height of the convolution kernel. In CBAM, the size of *h* × *w* is 7 × 7; after the CBAM-G attention module, the formula for the calculation amount is:

(7) $$GFLOS=\frac{C_{1}}{g}\times C_{2}\times (\frac{H-h}{S_{h}}\times \frac{W-w}{S_{w}})\times (h\times w)$$

In the above equation, g is the number of groups, and the size of *h* × *w* is 7 × 1. Therefore, after the CBAM-G module, the amount of computation will be reduced, and the convolution operation of multiple groups can improve the spatial perception. As such, the detection accuracy can be effectively improved.

### 3.2. Object-Contextual Feature Fusion Module

In semantic segmentation, the resolution used is full resolution, which is more suitable for small-scale and multi-scale object perception than object detection. In the contextual aggregation problem of semantic segmentation, the main idea of OCRNet [39] (Object-Contextual Representation) is to use the contextual features of a pixel point corresponding to an object to reinforce the features of that object. To address the unsatisfactory detection of multi-scale objects, we added an object-contextual feature fusion module [40] to provide more semantic information to the model and improve the perception of multi-scale objects. This module is equivalent to an attention module, which is a lightweight segmentation decoder that focuses on the connection between pixels and pixels in the corresponding object area. 

**Figure 6:**
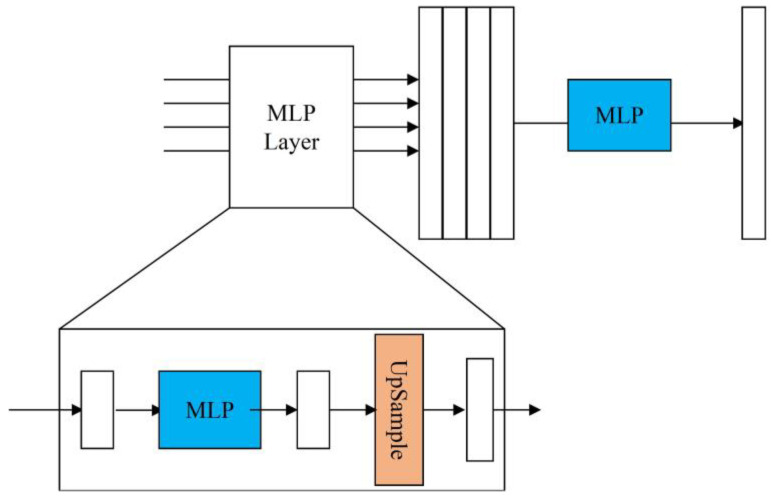
Object-contextual feature fusion module.

To improve the multi-scale object detection ability of the model, the results obtained after passing through the module were converted, and the pooling operation was performed first, and then the dot multiplication was performed with the category-aware channels in the detection heads of the three scales. Figure 6 displays the structural diagram of the object-contextual feature fusion module. 

The output layers that have gone through the backbone and neck networks are used as inputs and aggregated by the MLP operations, with the following primary process.

Each set of input features enters the MLP layer and passes through a linear layer that transforms the dimensionality of the input features to a fixed dimension.

(8) $${\hat{F}}_{i}=Linear(C_{i},C)(F_{i}),\forall i$$

In the formula, ${\hat{F}}_{i}$ is the output feature map, Linear is the linear layer operation, $C_{i}$ is the dimension of the feature channel, *C* is the set fixed dimension, $F_{i}$ is the input feature map, and *i* represents the ith group of feature maps.

Then, an up-sampling operation is performed using bilinear interpolation to unify the resolution of all input features to the resolution size of the first set.

(9) $${\hat{F}}_{i}=Upsample(\frac{W}{4}\times \frac{W}{4})({\hat{F}}_{i}),\forall i$$

Subsequently, all of the feature maps output after passing through the MLP layer are stitched together in the channel dimension to obtain the aggregated features.

(10) $$F=Linear(4C,C)(Concat({\hat{F}}_{i})),\forall i$$

Finally, the aggregated features are passed through the MLP layer again, and this time the MLP operation is implemented by 1 × 1 convolution to obtain the segmentation prediction. The number of feature dimensions is mapped to categories.

(11) $$M=Linear(C,N_{cls})(F)$$

The module branch has only four parts and contains six linear layers, so the number of parameters will increase slightly.

### 3.3. Improved IOU-LOSS

The loss calculation of YOLOX can be divided into localization loss, category loss, and confidence loss. The calculation of localization loss is related to the actual and prediction boxes. In the COCO [41] dataset, objects with a pixel area smaller than 32 × 32 are small objects. Small objects occupy less pixel area and carry less feature information. Therefore, fewer small object features are extracted when performing object detection, resulting in weaker feature representations.

The detection effect of YOLOX on a small object is not as good as that of a normal-sized object. The general formula for calculating *IOU* is:

(12) $$IOU=\frac{A\cap B}{A\cup B}$$

A represents the size of the actual box, while *B* denotes the size of the predicted box.

The *IOU* loss is calculated as follows:

(13) $$LOSS=1-IOU^{2}$$

To enhance the loss of the small object, a scale factor β is introduced in the *LOSS* calculation, which is calculated as:

(14) $$\beta =\frac{1-(\frac{gt}{100^{2}})}{mean(1-(\frac{gts}{100^{2}}))}$$

In the above formula, *gt* is the actual box area of a single object of a single image, *gts* is the actual box area sum of all objects of a single image, and *mean* represents the mean value calculation.

The improved IOU-LOSS calculation equation is:

(15) LOSS=LOSS×β

Therefore, when the object is smaller, its true value *gt* will also be smaller, while the ratio will be larger, and the percentage of the object loss becomes larger.

### 3.4. The Network Structure of CF-YOLOX

**Figure 7:**
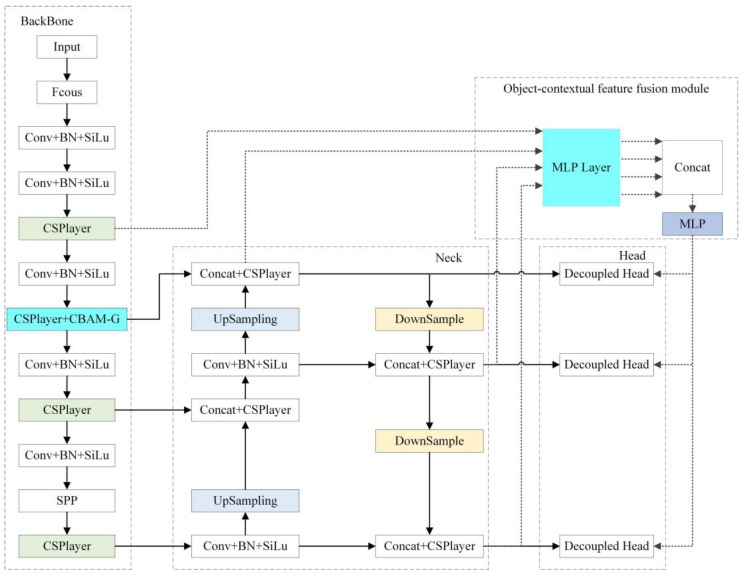
The network structure of CF-YOLOX.

Figure 7 depicts the CF-YOLOX network model after including the CBAM-G and object-contextual feature fusion modules.

The CF-YOLOX model can more accurately distinguish objects and backgrounds by introducing an attention mechanism, thereby improving the detection accuracy. By focusing on the critical features of the object region, the interference with irrelevant information can be reduced, thus, improving the robustness of the model. The backbone feature extraction ability of the network significantly impacts the model’s recognition effect. Therefore, the CBAM-G attention module was added to the backbone network to focus on the channels and spatial regions of interest, and the feature layer information was effectively extracted. Since the network shares the network weights, the effective feature layer of the next stage was also affected by the CBAM-G attention module.

Multi-scale feature fusion can improve the robustness of the model, making it less affected by factors such as illumination, rotation, and deformation. By fusing feature information of different scales, the dependence on a single scale can be reduced, thereby, improving the model’s generalization ability. The Neck network’s feature layer and the backbone network’s feature layer were jointly input into the object-contextual feature fusion module. The multi-scale feature was fed back to the prediction part to enhance the detection ability.

## 4. Experiments

### 4.1. Experimental Data and Details

This experiment used a server for training, Linux system, 64 GB, CPU using AMD EPYC 7601, GPU is NVIDIA GeForce RTX3090 with 24 GB of video memory. PyTorch framework was used, along with CUDA 11.3 for the computing acceleration.

We used the autonomous driving scene dataset KITTI as the training and testing dataset, which mainly labels objects such as vehicles and pedestrians. Some objects were obscured and truncated. The scene complexity could meet the data diversity requirements of autonomous driving detection models.

The KITTI dataset was labelled with eight categories, such as Car, Cyclist and Pedestrian, with 7481 images. The data samples were not evenly distributed, and the data categories were reclassified for testing. Firstly, Truck, Van and Tram were merged into the Car class; Person sitting was merged into the Pedestrian class; DontCare class and Misc class were ignored; and, finally, we merged the Car, Pedestrian and Cyclist classes. In this paper, the ratio of the training set to the test set was 9:1.

The training process and parameter settings were as follows: Mosaic and Mixup methods were used for data enhancement, the initial learning rate was 0.01, the self-adjustment method was set to cosine annealing, pre-trained backbone network weights were loaded, and a total of 150 cycles were trained.

### 4.2. Performance Evaluation

In the computer vision task, since the object detection task contained both classification and detection subtasks, its evaluation metrics needed to consider both the classification performance and the regression performance. *Recall*, *AP*, and *mAP* were selected as the evaluation metrics of the object detection algorithm. The calculation formulae of each evaluation metric are shown below.

(16) $$Recall=\frac{TP}{TP+FN}Re$$

(17) $$AP=\int_{1}^{n}P(R)dR\times 100\%$$

(18) $$mAP=\int_{1}^{n}\frac{AP_{1}+AP_{2}+\cdots +AP_{n}}{n}$$

In the above formula, *TP*: True Positive; *FN*: False Negative; *P*: Precision; *R*: Recall; *n*: categories.

Firstly, the ablation experiments were conducted to confirm the efficacy of the proposed CBAM-G module. The following five sets of experiments were designed to analyze the different contents. The model effect is shown in Table 1, with a “√” representing the method used for the model. The analysis of Table 1 shows that the mAP value of the original YOLOX network model was 90.61%, and the mAP value of Method 1 increased by 0.7% after adding the CBAM attention module. Method 2 was to consider the case that the aspect ratio of the image object changes after the image is compressed and stretched, and the spatial module of CBAM uses a convolutional kernel with a size of 7 × 7 to extract the features of the object, which improves the mAP value by 0.58% compared with Method 1. Method 3 verified the validity of Method 2. The vertical strip-shaped convolution kernel was for the case where the shape of the object becomes narrower and taller while using the horizontal strip-shaped convolution kernel to extract the features of the object; the accuracy is not improved but reduced, so it is effective to design a suitable convolution kernel for the aspect ratio of the picture object. Method 4 proposed the module grouping based on Method 2, which is the CBAM-G attention module proposed in this paper. Convolutional extraction of multiple groups can improve the global perception of attention to a greater extent, while grouping also reduces the computation and the number of parameters of the module. Using Method 4, the mAP value was improved by 0.66% compared with Method 2.

**Figure 8:**
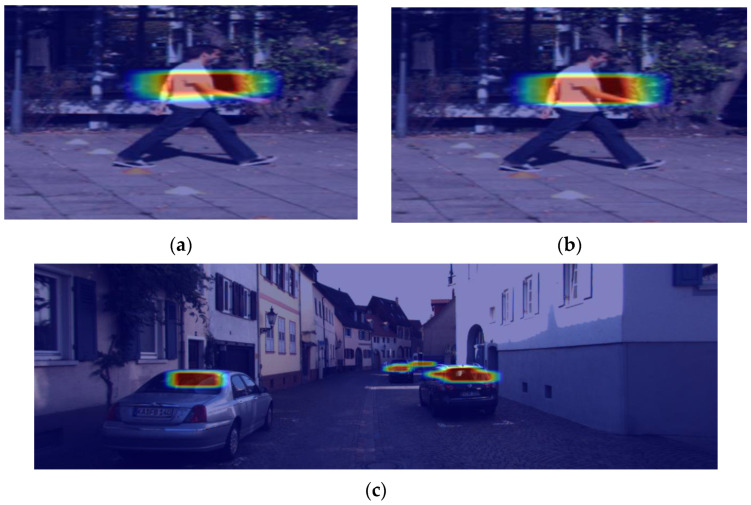

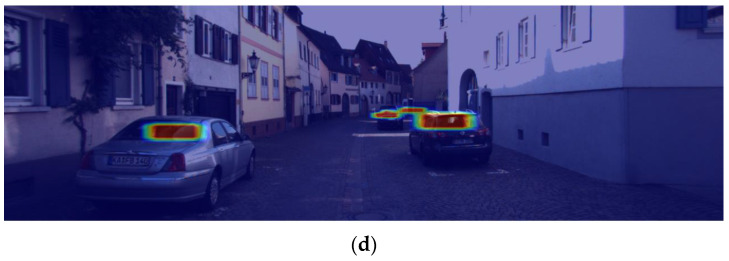
(**a**,**c**) Heat map of YOLOX model; (**b**,**d**) Heat map of method 4.

For a more visual comparison of the effect after the introduction of the CBAM-G module, a visual comparison of the heat map is performed in Figure 8.

As can be seen from the Figure 8, using Method 4 allowed the model to focus more on the region of interest and, thus, represent the salient features more clearly. This characteristic is advantageous for subsequently extracting the essential object features.

The CF-YOLOX model was enhanced by integrating the CBAM-G module, the object-contextual feature fusion module, and an improved IOU-LOSS computation method, which collectively improved the model’s detection accuracy from multiple perspectives. The performance comparison is shown in Table 2. A represents the original method, B represents the CBAM-G method, and C represents the object-contextual feature fusion method. Method D used improved IOU-LOSS based on Method C, and E represents the CF-YOLOX network model.

Table 2 illustrates that incorporating the CBAM-G module into the model does not significantly increase the parameters, yet it effectively improves the model’s mAP value by 1.94%. Adding the object-contextual feature fusion module increased the parameters by a small amount, but the mAP value could be improved by 2.0%; after introducing the improved IOU-LOSS calculation method, the mAP value increased to 93.07%.

Table 3 shows the recall values for the different categories of Methods A–E.

Based on the information presented in Table 3, it can be observed that the recall values of Car, Cyclist, and Pedestrian were improved to some extent after adding different modules or methods.

To further demonstrate the superiority of the proposed model, experimental comparisons were conducted with different models on the KITTI dataset, all of which were of size “S” or lightweight networks, as shown in Table 4. The mAP value of YOLOv6 [29], YOLOv7-tiny [30], and YOLOX were similar, but YOLOv6 had a larger number of parameters, reaching 18.50M; the CF-YOLOX model had the highest mAP value and a moderate number of parameters, which had a more productive detection performance.

We also used some data from the BDD-100K dataset for experimental comparison, a total of 10,000 pictures; the ratio of the training set to the test set was 8:2. The BDD100K data set had a total of ten categories, namely: bus, traffic light, traffic sign, person, bike, truck, motor, car, train, and rider. Considering that the sample distribution of the data set was not uniform, it was necessary to reclassify the sample categories. The four categories of truck, train, bus, and car were combined into the car category, and the three categories of motor, bike, and rider were combined into the rider category. The final sample categories were: car, traffic light, traffic sign, person, and rider. Based on this data set, different algorithms were trained and tested, as shown in Table 5.

The scene of the BDD100k dataset was complex, so the mAP value of the model may be lower, but the model’s generalization ability will be increased. The calculation and inference time of the CF-YOLOX model was the highest, but it was still realtime, reaching 13.4 ms, which was only 0.4ms lower than the original YOLOX model. It is advisable to trade time for accuracy without affecting the realtime requirements of the model. More elevated precision detection models can be more capable of perceiving the environment. The mAP values of YOLOv5 [42], YOLOv6, and YOLOv7-tiny were lower, which was determined by the model structure itself. The lower calculation and inference time can make the model better suited for embedded devices. The “car” category had more samples in the data set. The object shape and texture were relatively clear, so the AP values obtained by each model for the car category detection were relatively similar, and the value obtained by the CF-YOLOX model was the highest, reaching 74.72%. For other categories of the test results, due to the difference of the model and the complexity of the sample environment, the difference was relatively large.

**Figure 9:**
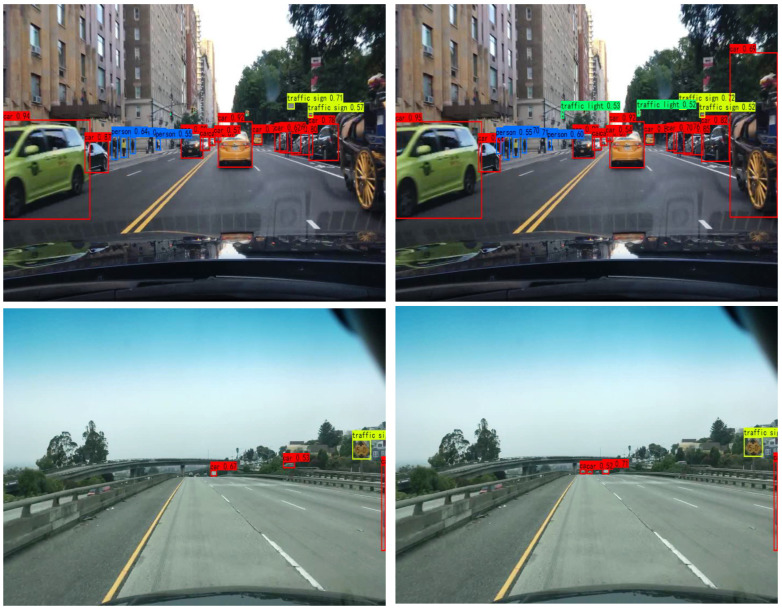

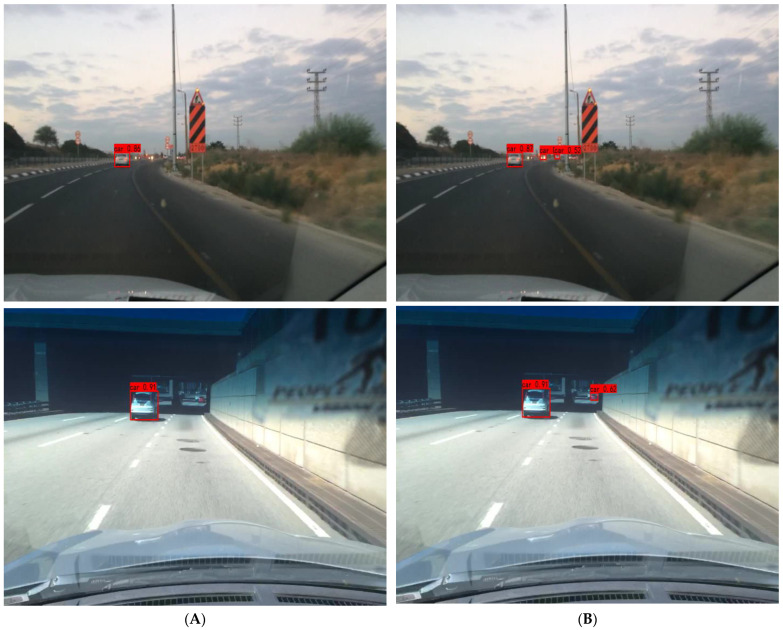
The detection effect of different scenes: (**A**) is the detection effect of YOLOX; (**B**) is the detection effect of CF-YOLOX.

Figure 9 shows the detection effect of the YOLOX model and the CF-YOLOX model in different scenarios. In the first row of Figure 9, the YOLOX model missed the traffic light in the distance, while the CF-YOLOX model could detect it, indicating that the CF-YOLOX model had a more robust ability to detect small objects in the distance. In the second, third, and fourth rows, the vehicle object located in the distance can be regarded as a small object, and the original model missed the detection of these smaller objects. Since the CF-YOLOX model had added an object-contextual feature fusion module with multi-scale feature fusion characteristics and an improved IOU-LOSS that can improve the loss of small objects, the CF-YOLOX model had a preferable detection effect on these small objects. 

## 5. Conclusions

This paper proposed a multi-scale object detection model based on YOLOX to solve the problems of missed detection and inaccurate recognition in multi-scale object detection in complex scenes of autonomous driving. We added the proposed CBAM-G to the YOLOX model, which can focus on the critical feature information of the object. We proposed an object-contextual feature fusion module to obtain more semantic information and improve the object perception at different scales. The output of this module was input to the detection head together with the output feature map of the neck network to improve the object detection effect at different scales. Finally, we proposed an improved IOU-LOSS calculation method, which is beneficial to enhance the detection ability of small objects. We conducted comparative experiments on the KITTI dataset and the BDD100K dataset. The proposed model had the highest mAP value, indicating that the model has wide applicability and can meet the detection requirements of recognizing objects in autonomous driving scenarios. In future research, we will continue to study multi-scale object detection, while considering the issue of lightweight to be preferable to apply in practical application scenarios.

## Figures and Tables

**Table 1:** Ablation experiment of CBAM-G.

Method	CBAM	7 × 1	1 × 7	Group	mAP@0.5/%
YOLOX					90.61
1	√				91.31
2	√	√			91.89
3	√		√		90.94
4	√	√		√	92.55

**Table 2:** The AP values of different models.

Method	Parameter/M	Car/%	Cyclist/%	Pedestrian/%	mAP@0.5/%
A	8.938	95.61	93.25	83.13	90.61
B	8.939	96.27	95.53	85.85	92.55
C	9.448	96.37	95.77	85.70	92.61
D	9.449	96.54	94.77	87.45	92.92
E	9.449	96.76	95.85	86.61	93.07

**Table 3:** Recall value for different categories of methods A to E.

Method	Car/%	Cyclist/%	Pedestrian/%
A	92.08	89.12	74.54
B	93.61	91.16	77.78
C	93.58	91.84	77.55
D	93.64	91.84	78.94
E	93.95	91.84	80.32

**Table 4:** Comparison of different models on the KITTI dataset.

Model	AP@0.5/%			mAP@.5/%	Parameter/M
	Car/%	Cyclist/%	Pedestrian/%		
YOLOX	95.61	93.25	83.13	90.61	8.94
YOLOv5	97.80	95.00	84.90	92.50	7.01
YOLOv6	97.10	91.90	82.80	90.40	18.50
YOLOv7-tiny	96.40	94.00	81.40	90.60	6.01
CF-YOLOX	96.76	95.85	86.61	93.07	9.49

**Table 5:** Comparison of different models on the BDD100K dataset.

Model	AP@0.5 (%)					mAP@0.5/%	Inference Time/ms
	Car/%	Person/%	Rider/%	Traffic Light/%	Traffic Sign/%		
YOLOX	73.58	57.47	39.33	57.17	57.96	57.10	12.9
YOLOv5	71.50	50.1	37.7	49.1	53.50	52.40	3.4
YOLOv6	71.2	47.00	25.90	40.80	49.50	46.90	3.15
YOLOv7-tiny	72.6	51.7	39.1	43.8	50.00	51.40	2.6
CF-YOLOX	74.72	57.60	40.89	57.69	59.82	58.15	13.3

## Data Availability

The data presented in this study are available on request from the corresponding author.
